# Bridging the gap: a qualitative process evaluation from the perspectives of healthcare professionals of an audit-and-feedback-based intervention to improve transition to adult care for young people living with type 1 diabetes

**DOI:** 10.1186/s12913-024-11734-1

**Published:** 2024-10-23

**Authors:** Janet A. Parsons, Jannah Wigle, Ian Zenlea, Noah Ivers, Geetha Mukerji, Alanna Landry, Zubin Punthakee, Cheril L. Clarson, Rayzel Shulman

**Affiliations:** 1 Department of Occupational Science and Occupational Therapy, Temerty Faculty of Medicine, University of Toronto, 160-500 University Ave., Toronto, ON M5G 1V7 Canada; 2 Applied Health Research Centre, Li Ka Shing Knowledge Institute, St. Michael’s Hospital, Unity Health Toronto, Toronto, ON Canada; 3 Institute for Better Health, Trillium Health Partners, Mississauga, ON Canada; 4 Institute of Health Policy Management & Evaluation, Dalla Lana School of Public Health, University of Toronto, Toronto, Canada; 5 Department of Paediatrics, Temerty Faculty of Medicine, University of Toronto, Toronto, ON Canada; 6 Women’s College Institute for Health System Solutions and Virtual Care, and Department of Family Medicine, Women’s College Hospital, Toronto, Canada; 7 Women’s College Hospital Institute for Health Systems Solutions and Virtual Care, Women’s College Hospital, Toronto, ON Canada; 8 Institute of Health Policy, Management and Evaluation, University of Toronto, Toronto, ON Canada; 9 Department of Medicine, Temerty Faculty of Medicine, University of Toronto, Toronto, ON Canada; 10 Department of Pediatrics, Oak Valley Health, Markham, ON Canada; 11 Department of Medicine, McMaster University, Hamilton, ON Canada; 12 Department of Paediatrics, University of Western Ontario, London, ON Canada; 13 Division of Endocrinology, The Hospital for Sick Children, Sickkids Research Institute, Toronto, Canada; 14 Department of Paediatrics, University of Toronto, Toronto, Canada; 15 Institute of Medical Science, University of Toronto, Toronto, ON Canada

**Keywords:** Type 1 diabetes mellitus, Adolescence, Transitions in care, Qualitative methods, Process evaluation, Quality improvement

## Abstract

**Background:**

The transition from pediatric to adult care is a vulnerable time for young people living with type 1 diabetes (T1D). Bridging the Gap (BTG) is an audit-and-feedback (AF) intervention aimed at improving both transitions-in-care processes and diabetes management in the year following transition. As part of BTG, we conducted a qualitative process evaluation to understand: (a) what was implemented and how; and (b) the contextual factors (micro-, meso- and macro-) that affected implementation, outcomes and study processes.

**Methods:**

Using qualitative descriptive methodology, interviews were conducted with 13 healthcare professionals (HCPs) delivering diabetes care to transitioning youth. Participants were asked about their experiences of BTG study processes and feedback tools, the quality improvement (QI) initiatives implemented at their site, and potential spread and scale. Interviews also explored the impacts of COVID-19 on transition care and study processes and results.

**Results:**

Five key themes were identified. Participants’ *reflections on the BTG study design* indicated they appreciated its flexible, site-specific approach to QI, which they saw as crucial to the success of their initiatives. *Engagement with feedback reports and other study resources* provided comparative, site-specific data. Participants described *the challenges posed by the COVID-19 pandemic* and its impacts on patients, care provision and study implementation. Their *site-specific QI initiatives* resulted in changes to their transition practices. Finally, participants commented on how BTG and its processes *fostered a community of practice (CoP)* between sites, resulting in new opportunities to collaborate and share experiences.

**Conclusions:**

BTG resulted in a CoP among practitioners delivering transition care to youth with T1D, which could be scaled up to promote a learning health system in pediatric diabetes care. Qualitative process evaluation is a useful tool for understanding how contextual factors affect the implementation and outcomes of complex QI interventions.

**Supplementary Information:**

The online version contains supplementary material available at 10.1186/s12913-024-11734-1.

## Introduction

Adolescence is a vulnerable period for people living with type 1 diabetes (T1D) [1, 2]. As young people mature, they take on increasing responsibility for diabetes self-management, which must be balanced with the competing demands of school, work, and relationships [2]. Glycemic management frequently deteriorates during adolescence and early adulthood [3], augmenting the risk of future acute and chronic diabetes complications [4, 5]. The transition period from pediatric to adult care is when young persons living with diabetes may be lost to clinical follow-up [6–8], which is also associated with poorer self-management [2, 9]. Ensuring young adults connect with new adult healthcare providers (HCPs) and remain connected can be challenging, especially when they are moving away from home for the first time, starting work, or embarking on post-secondary education [2, 10].

The *Bridging the Gap* (BTG) study was undertaken to develop and test an audit-and-feedback-(AF) based intervention aimed at improving both transitions-in-care processes and diabetes management in the year following the transfer from pediatric to adult care [6, 11]. AF involves measuring quality of care and feedback by comparing results to accepted standards and/or peer performance [12]. BTG sought to implement the evidence-based ‘Got Transition’ approach [13], which outlines ‘key ingredients’ to successful transition care and was used to inform quality improvement (QI) initiatives by local HCP teams at each of the five study sites [14]. The BTG study itself has been described in detail elsewhere [11].

A process evaluation was conducted as part of BTG. The aims for the overarching process evaluation included understanding: what was implemented and how; whether the intervention was received as intended (intervention fidelity); mechanisms by which the interventions brought about change; and how micro-, meso- and macro-level contexts affected BTG’s implementation and outcomes [15]. A separate paper focuses on insights about the adaptation of and fidelity to the BTG intervention by the research team and participating sites [16]. Here, we report on a qualitative sub-study nested within the process evaluation, which is focused on the results from a series of *post-implementation qualitative interview*s. The aim of our qualitative investigation was to explore contextual considerations that impacted the study (both processes and outcomes) through the perspectives of HCPs who contributed to implementing BTG at their institution. Our specific objectives were to understand HCPs’ perspectives as they related to: (a) their experiences of designing and implementing QI initiatives at their site; (b) their experiences of BTG study processes/procedures; and (c) challenges to implementation encountered. HCP participants also reflected on the effects of the COVID-19 pandemic and resulting adaptations in transition care. BTG was designed before the pandemic’s onset; therefore there were unexpected consequences for study implementation.

Our study design was informed by principles of formative, qualitative program evaluation which tend to be context-specific, process-oriented, and rely on approaches grounded in the interpretivist paradigm [17]. We also recognized that organizational context [18] would be centrally important to improving transition practices.

## Methods

### Setting and study design

The broader BTG study used a pre-post design to test the effectiveness of an AF-based intervention in pediatric diabetes clinics aimed at improving glycemic management among young people living with T1D in the first year after transfer from pediatric to adult care [11]. BTG was implemented across five different urban hospital sites in southern Ontario, Canada – each with a pediatric clinic supporting adolescents with T1D and their families and experienced in providing transition care. The sites included three tertiary and two large community-based clinics. The multi-disciplinary HCPs at these sites prepared patients and their families to transition to adult care. Each site had its transition-related practices and processes of care (which varied between sites) before the initiation of the study, and they subsequently developed unique quality improvement (QI) interventions tailored to their specific contexts. Our process evaluation examined these local practice-level QI interventions, employing a qualitative descriptive design and informed by realist evaluation approaches [18–20]; this methodology is well-suited to exploring HCPs’ experiences within their multidisciplinary teams as they developed practice innovations. While we had initially planned to conduct post-implementation focus groups with teams at each study site [11], COVID-19 pandemic-related considerations (e.g., staff availability due to redeployment, shifts in practice, staff shortages, time constraints) made individual interviews more feasible.

Ethics approval for this study was granted by the Clinical Trials Ontario (CTO) Streamlined Research Ethics Review System for four out of five study sites. CTO research sites participating in a multi-site research study undergo a single ethics review. The Trillium Health Partners Research Ethics Board also granted ethics approval. All participants provided written informed consent.

### BTG interventions

Diabetes teams developed QI interventions at each of the five study sites. Multi-disciplinary teams at each site identified the priorities for their team and this was kept deliberately flexible. The sites were free to determine the aim of their QI initiative, guided by the Got Transition guideline and transition care performance feedback reports [21]. In addition to feedback reports, BTG included a series of webinars, allowing providers to have facilitated conversations about the reports and their progress to date. Site-specific feedback reports provided information on patient participant characteristics, transition experience measures, and patient transition outcomes. Feedback reports were issued at baseline (before implementing their interventions) and before webinars. Sites were guided to develop their QI initiatives to meet specific needs identified as priorities for a given site. Webinars were conducted across sites and facilitated by senior members of the study team (IZ and RS). Quorum, an online community dedicated to improving the quality of health care across Ontario, was used to host a private forum for providers participating in BTG. Participants could view transition and QI resources posted by the study team. There was also an opportunity to interact with other provider participants about progress of their QI initiatives via a discussion board. A detailed description of the site-specific interventions is outlined elsewhere [16].

### Participant recruitment

Interview participants were recruited from across all five sites. No individuals declined to participate. Interviews were conducted in May 2022. An email invitation to participate in the process evaluation (focus group or interview) was sent by the site Division Heads to site HCPs, with potential participants instructed to contact the research team directly if interested in the study. All participants provided individual interviews and no focus groups occurred for the reasons outlined above (see Setting and Study Design).

### Data collection

Individual in-depth interviews were conducted by telephone or Zoom, based on participant preference. Interviews were conducted by team members with expertise in qualitative methodology (JW and JAP). All interviews were digitally audio-recorded and transcribed. A semi-structured interview guide (Supplementary File: Interview Guide) was developed specifically for this study, covering topics such as participants’ experiences of BTG study processes and feedback tools, reflections on the QI initiatives implemented, and insights regarding potential spread and scale. Interviews were designed to gain insights into what it was like to be part of BTG (including study processes common across sites), but also to elicit reflections on the QI initiatives implemented at their specific site, and any successes or challenges encountered.

This study began before the COVID-19 pandemic and continued through it. Like many research projects, BTG’s implementation was profoundly impacted by the pandemic and resulting changes to practice and research. It also significantly impacted the lives of adolescents with T1D, their families, and their social networks. Therefore, the interviews also explored how COVID-19 impacted transition care and the study’s implementation and results.

### Data analysis

Using a qualitative descriptive approach [19, 20], our analysis combined inductive and deductive strategies [22]; while we began with exploring the data, examining it for regularities and patterns, we also examined the data from the lens of implementation science to identify aspects of the study that were of interest to the research team from the outset (e.g., how participants were interpreting and acting upon the team reports) – in keeping with a deductive approach [23]. Two researchers with qualitative research expertise led the data analysis. JW, an experienced qualitative researcher, descriptively coded all the transcripts, and JAP (a senior qualitative researcher) coded a subset of the transcripts. The emerging analytic framework was presented to the broader study team for discussion and feedback.

We recognize that reflexivity is important in qualitative research [22]. Critically interrogating our research interests and professional identities shaped our approach to qualitative process evaluation, research questions, as well as analysis/interpretation of the data generated [24]. Our understanding of challenges encountered during emerging adulthood and gaps in diabetes transition care [1, 2] were informed by our professional positions as clinician scientists (IZ, NI, RS), rehabilitation scientist (JAP) and critical qualitative health researchers (JAP, JW). Our diverse experiences working with young people and conducting research on primary care, quality improvement, implementation science, adolescence/ emerging adulthood, and diabetes care also shaped the research process.

## Results

Interviews were conducted with 13 healthcare providers delivering diabetes care to transitioning youth across all five study sites. Participants included pediatric endocrinologists, nurses, and dietitians. Most participants (*n* = 12) identified as female. Interviews lasted between 26 and 53 min (median 43 min).

Participants offered in-depth accounts of their experiences developing QI initiatives at their sites. Their experiences participating in the BTG study were also entangled with the co-occurrence of the pandemic. With that in mind, we organize our results into five key themes: *reflections on the BTG study design; engagement with feedback reports*,* webinars and discussion forum*; *challenges posed by the COVID-19 pandemic; site-specific QI initiatives;* and *developing a community of practice.*

### Reflections on the BTG study design

Most participants highlighted the importance and value of the BTG study and its focus on transition. Several participants indicated that BTG helped teams critically (re)consider their transition-related practices and improve support for young patients during their transition to adult care.

*“One of the most helpful things about the Bridging the Gap [study] was…the microscope on transition and really giving us that push to do some of the things that we really should be doing and kind of reflecting on our practice for that age [group].” (P04)*.

Participating in the study was portrayed as an opportunity to reflect on their current practices. The study provided the time and incentive to refine their approaches to transition, to make their transition processes more person- and family-centred, and to engage in follow-up – to ensure that the transition had been accomplished as planned. As one participant said,

“*I think we’ve just been a lot more understanding [of] what the family’s needs are and then thinking about making the resource fit to the family. I think what’s also going well is*,* we’ve all identified that it’s a period of time where things get lost.” (P13)*.

Participants indicated a commitment to improving the quality of not only their own local practices, but also to learning from experiences at other sites [25].

Participants appreciated the study’s flexible, site-specific approach to QI, which they characterized as crucial to the success of their initiatives. Most participants underscored that if the study had aimed to introduce a standardized intervention across all five study sites, it might not have been well-suited to a given site’s existing structures or systems (e.g., electronic medical record (EMR) systems, resources/staff available). They stressed that there is no “one-size-fits-all” approach to transition care.

*“I think giving the sites the autonomy to make decisions around which aspects of that transition process they wanted to target was the right way to do it.” (P11)*.

*“But it was interesting to see how every hospital was in a different stage of thinking about transition. Obviously*,* I think everybody was invested and interested*,* but people were just at different stages of the process…So I think it’s great to leave it open and flexible for every hospital to do what works for them.” (P08)*.

Several participants expressed that the study’s flexible approach reflected their unique contexts (including social determinants, populations) and celebrated the teams’/sites’ creativity:

*“[The] social determinants of health may be different in some areas or the capacity of a centre or focus of that centre may be totally different. So I think that was a good approach*,* to allow the [site] researchers to determine what should be priority.” (P12)*.

Participants commented that if a more prescriptive or standardized approach across sites had been adopted, ownership and sustainability of the initiatives might have been undermined. Teams’ commitment to the process was enhanced by permitting them to develop locally relevant QI initiatives and tailor them to their own practice contexts.

Despite overall positive feedback on BTG’s approach, some concerns were shared that site-specific initiatives may have duplicated efforts across sites or limited cross-site exchange of knowledge and resources. The open-ended nature of the study was described as an obstacle for some sites, and identifying aim statements was sometimes a lengthy and challenging exercise.

“*I can say that we had a tough time engaging in this study because it was so open-ended. So*,* at the beginning*,* we weren’t really sure what we were supposed to be doing…I think our aims and goals were probably not very clear because we had so many other things going … at the start of the study…”(P06)*.

Overall, however, participants appreciated the BTG study’s goals, design and implementation, and the ability to adapt their QI initiatives as needed.

### Engagement with BTG feedback reports, webinars and online discussion forum

Before the BTG study, sites did not have systematic approaches to measuring/evaluating transition processes. Interviewees emphasized the reports were important tools to provide site-specific feedback and to compare data and progress across sites. Teams regularly used the reports to inform both formal (e.g., existing weekly departmental huddles/meetings) and informal discussions (e.g., to provide study updates, brainstorm concepts and introduce initiatives). Participants said this allowed for increased engagement from other practitioners within their departments who were not actively part of BTG. Several participants reported being pleased to see that they were performing well despite encountering significant challenges to study implementation.

*“It’s nice to have the site-specific feedback and then the feedback for all the different sites involved…you were able to see how things went and compared to the year before and see how your goals have changed… it was helpful too*,* because the specific feedback we received*,* we were able to share with the remaining practitioners in the department and so it felt like it wasn’t just the people directly involved in the transition process that were able to hear the feedback and give input.” (P02)*

*“I really liked hearing what we were doing well or not doing well because that’s something that we would never have known before….” (P08)*.

Participants portrayed the regular feedback as a motivating factor to continue to improve care delivery for young patients with diabetes and their families.

Feedback reports also represented a novel opportunity to compare performance and understand approaches across sites. As one participant said, “*it’s always better to have multiple brains than one and think about how they’re doing it.” (P01)*. This sparked further reflection on site-specific practices and the importance of local context, while engendering ideas for future QI initiatives.

Several participants reported being less familiar with the reports, sometimes because they were less involved in the BTG study, joined the team late, or because of time constraints. Whereas others highlighted that key findings from the feedback reports were difficult to interpret and/or act upon. Some suggested that additional comparisons, whether based on geography or hospital type (e.g., academic versus community hospital), might spark further interpretations and ideas for strategies to improve transition care in their context.

Most study participants indicated they had attended one or more webinars over the course of the study and found them informative and engaging. While times/dates for the webinars suited most participants, some said they conflicted with their clinic schedules and had trouble fitting them into their busy schedules.

Many participants reported limited engagement with the online discussion forum (Quorum), which was reinforced by others’ limited engagement. Time constraints, having to proactively look at Quorum and being overloaded with other online commitments/meetings during the pandemic were also identified as potential reasons for not engaging with this resource.

### Difficult work in challenging times: transforming transition practices in a pandemic

BTG’s findings cannot be disentangled from the co-occurrence of the pandemic. Globally, the pandemic disrupted research and clinical practice in profound ways and BTG was no exception. The pandemic’s onset coincided with site teams initiating their QI interventions. Participants reflected at length on COVID-19’s impact/s on both their clinical lives and study implementation.

#### Impacts on study implementation

Implementing BTG study-related initiatives may have been delayed and/or not prioritized amidst competing demands generated by the pandemic. Many participants emphasized that the pandemic contributed to significant upheaval in terms of staffing (e.g., staff redeployment to other clinical services), less dedicated time and resources to devote to the study, and changes to transition care delivery in some institutions.

*“I feel like the emphasis on it [BTG] was probably lessened*,* given that this plan was started in the beginning of 2020…when COVID sort of first started*,* we went completely virtual and our [clinical staff] visits have actually remained almost entirely virtual*,* until recently. I think that working on our virtual care and ensuring that those systems were in place probably took priority over the continuation of that transition readiness tool.” (P06)*.

*“There were many*,* many real-world factors that impacted our ability to be successful with implementing the project. Our team still consistently attended the webinars*,* we still completed their reports ….* [but] *we were struggling during the pandemic*,* our clinic*,* with staffing and operations.” (P11)*.

This quote exemplifies participants’ commitment to completing the BTG study despite the challenges presented by the pandemic. However, participants also expressed that the use of online meetings, webinars and sharing of electronic copies of the feedback report came at a time when there was considerable “screen fatigue” (P01). This shift from in-person to virtual work (in all facets of their roles) affected some participants’ engagement with the study. Another trend was a shift to physicians leading virtual visits, whereas a multidisciplinary approach was the norm pre-pandemic. This was problematic for the study since BTG was premised on a multidisciplinary approach to QI.

#### Impacts on clinical practice

The same clinical practice changes outlined above that impacted study implementation also affected some sites’ capacities to provide transition care for youth. The sudden shift to virtual care in some instances removed the opportunity for HCPs to “say goodbye” to transitioning patients. Some providers said they missed the one-on-one engagement with youth in the clinic setting. They also remarked on a lack of privacy from parents/caregivers during virtual appointments with youth, which could limit opportunities for covering sensitive, transition-related issues, including discussions about alcohol, drugs, sexual activity, or pregnancy.

The pandemic’s impact on young patients’ mental health was also cited as a concern, which often took priority over planning for transition.

*“There was a huge amount of mental health issues during COVID that increased*,* and so I think that that always makes it more challenging to talk about your transition topics*,* when there’s a more pressing mental health issue that needs to be addressed.” (P02)*.

While participants talked at length about the negative impacts of COVID-19 on their usual care practices, they also identified potential benefits. For example, some participants said it encouraged young people to take on more responsibility for self-managing their diabetes. In addition, the shift to virtual care increased accessibility for some youth.

*“In some ways*,* [virtual visits] made care more accessible*,* obviously*,* because there’s not too many excuses people can make for not making a virtual appointment as opposed to like an appointment downtown where they have to drive and weather and park and all those kinds of things.*” *(P09)*.

### Site-specific QI initiatives: adapting practices to local contexts

Many participants outlined changes to their transition practices, including tools, appointments and approaches, due to their involvement in the BTG study. BTG helped them to prioritize a focus on transition.

*“[Discussion of BTG feedback reports] serves as a reminder to everybody to kind of make sure that they have transition … [as]one of the things that they’re thinking about with every one of the youth. And I think that one of the things we spoke about was basically*,* how do we make sure that we capture every one of these children and ensure that they’re taken through the most important part of the transition curriculum?” (P02)*.

Another participant commented that they specifically changed some of their administrative practices to ensure they communicated with the transitioning patient directly.

*“So one of the things I’ve changed personally in my own practice is to make sure I get my admin*,* if we don’t have it in the chart*,* to get an email address and a cell phone number for the 18-year-old. So that gets put into the referral letter that I send to the adult [clinic]…That’s one of the comments that came up in audit-and-feedback sessions*,* was that the kids didn’t report it*,* that they didn’t know the name or contact information of their new doctor*,* and they probably received it*,* but it would have been received either by their parent or just to their home address as opposed to being directed at them.” (P01)*.

The interventions developed helped to formalize strategies and/or practices, enabling *“a much more structured approach to discussing transition topics and more time in the clinical setting to do this.” (P09).* Some participants commented that it fostered a more ‘standardized’ process with which to approach transitions. Integrating the site teams’ interventions into existing clinical practices and structures (such as programming prompts about transition in their EMR) helped facilitate improvements.

Participants commented on how their initiatives were tied to struggles they’d encountered with transition care at their facilities. While the site-specific initiatives varied, the overall benefit of focusing on transition practices, reflecting on them within a collaborative framework, developing new tools and procedures to improve transition for their patients – and then assessing outcomes post-implementation --- were all seen as helping them improve the supports they offered patients and their families.

### Developing a community of practice: present and future opportunities

Many participants appreciated the unique opportunity BTG represented to be part of a ***“****learning community****”****(P08)*, to formally connect with diabetes care providers at other sites and learn about other sites’ QI initiatives – an essential ingredient to communities of practices (CoPs) [25] They valued exchanging knowledge and discussing challenges experienced in diabetes transition care.

*“I thought that [the webinars] were great. It’s really nice to actually have a dedicated time to speak about bridging the gap in transition with all the different sites involved. What I really liked was that it’s sites that we kind of informally work with all the time*,* yet don’t get a lot of opportunity to actually speak face-to-face or at least by Zoom … we went around and everybody gave a little update from each one of the sites …. it was a really nice way to collaborate in real time.” (P02)*.

Learning from ‘what was already working’ at other sites was seen as helpful to improving their own site’s practice/s. Feedback reports offered an opportunity to compare performance, share experiences, and learn from other programs, which they had never been able to do before.

Despite the extensive challenges presented by COVID-19, multiple participants said they were impressed by other centres’ accomplishments, their creativity, and the impressive commitment amongst BTG sites to improve transition experiences for young adults.

*“I was very sad that we had not moved forward very quickly and not really done much*,* honestly…but it was encouraging to see what some other centres had been able to do. It’s pretty creative and it was a bit inspiring. And it makes you want to step up and do better for sure because you want to be a good centre. You want to have the best quality of care that you can have and you want to prep your kids the best you can.” (P10)*.

In this way, the reports and webinars helped to build the CoP since site teams could learn what other programs were doing.

*“It was clear that COVID has had lots of effects on the transition process*,* and so I’m sure that some of the study results maybe reflect that a little bit…my experience with [intervention] has been quite good…like we started with no formalized transition process*,* and we really are referring a lot of the eligible patients to the transition-dedicated visit. But I think that there’s still a lot of work to do.” (P02)*.

They emphasized future opportunities to build upon the BTG study to expand the CoP to include additional pediatric sites and adult endocrinology teams. Participants expressed interest in increasing connections between youth and adult HCPs to improve communication and ease the transition for young adults.

*“I implore the community*,* the smaller centres and those who have an adult and a pediatric centre to do stuff together. I think like have lunch and learns together*,* have conversations about this outside of patient care ….” (P13)*.

Suggestions included holding discussions between pediatric and adult teams (perhaps using some of the checklists/flow sheet tools developed during BTG) and having youth meet their new adult providers before the formal transition to adult care. Improving transition curricula and follow-up assessments to track youth post-transition were also recommended. Greater involvement of youth themselves in identifying gaps in transition education and in designing new tools was also suggested. To expand the CoP, participants recommended a mentorship approach whereby BTG sites could work with new pediatric centres looking to introduce QI initiatives. As such, our participants spoke to systems thinking, which is essential to establishing a CoP.

## Discussion

This investigation was designed as a qualitative process evaluation to understand how BTG contributed to practice change at the study sites, the experiences of participants in implementing their QI interventions, and the contextual features that impacted their engagement with the study. Engagement in the study offered HCPs working with youth transitioning from pediatric to adult diabetes care the opportunity to critically and consciously reflect upon their care delivery practices and policies. BTG helped the diabetes care teams/sites prioritize the critical transition period between pediatric and adult care by exposing and addressing gaps, and championing novel tools, resources, and systems. The feedback reports offered teams the opportunity to evaluate changes in their care delivery. Participants valued the chance to reflect upon their current transition practices, identify ways to tailor QI initiatives to their specific site contexts and track their progress via the feedback reports. The online webinars brought teams together to share experiences and collaborate in ways they had previously been unable to. The study also highlights the importance of building capacity between tertiary and community-care centers around transition practices, which is often overlooked in research designs. Despite largely positive feedback on the study’s goals and processes, challenges were encountered. Perhaps the biggest of these related to the COVID-19 pandemic, which caused unprecedented shifts in practice and research at every site. Staffing shortages and the shift to virtual care were often cited as barriers to conducting study activities. This extended to participants’ limited engagement with the Quorum online discussion forum, which was attributed to a lack of time for engagement secondary to short staffing, feeling overloaded with online meetings during the pandemic, not seeing others using it, and screen fatigue. In contrast, the webinars were generally well-attended with all study sites represented at each session [16]. However attendance varied and this could have been because of changes in staff availability and changing team composition over the two years of implementing BTG [16]. Whether and how the BTG intervention worked as intended (fidelity and adaptation) is reported elsewhere [16].

Our results document the building of a CoP on transition care in diabetes within and between study sites. Attending to contextual barriers and facilitators is essential to understanding the development of CoPs [26]. Participants spoke about how study processes enhanced communication, supported team learning, fostered organizational change, and aided systems thinking [27]. The informal interactions and reflections prompted by receiving the reports were seen as significant opportunities to consider what they were doing well and what might need improving in keeping with the principles of a learning organization – a feature of CoPs [27]. CoPs are “groups of people who share a concern, a set of problems … and who deepen their knowledge and expertise in this area by interacting on an ongoing basis” [28]. There is extensive literature demonstrating how CoPs can lead to healthcare improvements [25, 29, 30] and our results outlined several critical ways in which BTG fostered a new CoP. BTG’s focus on relational learning and between-site interaction, sharing information and knowledge, documenting practice changes over time, and attending to context (organizational and beyond) were emphasized [31].

There has been increasing uptake of CoPs in recent years, typically as ways to improve knowledge exchange and promote practice improvements [26]. While some CoPs are purposefully established, others develop less formally (as was the case for our study) [25]. CoPs enhance knowledge sharing, inspire innovation, and facilitate team learning [27]. Grounded in issues of local service delivery that matter to providers, CoPs help to foster practice improvements and organizational and system-level change [27]. Because CoPs span multiple organizations (as happened in BTG) they may be well-positioned to inform learning health systems [26, 32], whereby practice improvements are embedded into care delivery and new knowledge is generated through that delivery process [32].

The onset of the COVID-19 pandemic early on during BTG’s implementation posed unique challenges to the research team and all the participating study sites. Shifting priorities, staff changes and redeployment, and changes to clinical practices (e.g., shift to virtual appointments) required clinical staff and researchers to pivot their practices to adapt to the public health emergency. Because of this, participants spoke at length about how COVID-19 had impacted their transition practices and how this affected the rollout of the study itself.

### Recommendations

This study generated noteworthy recommendations from the interviewees. The CoP established across study sites was highly valued and praised by participants, and opportunities to maintain inter-institutional networks and the relationships forged should be pursued. Increased sharing of resources (e.g., flow sheets, checklists, and education resources) was highlighted by many participants as an essential improvement engendered by BTG. This included using transition flow sheets to share between pediatric and adult care teams so that issues could be tracked over time.

Opportunities to validate study tools (e.g., transition-tracking flow sheets) were identified as a future area for research. Increased collaboration with site-specific QI initiatives and resources (e.g., QI coordinators) was identified as a potential opportunity to alleviate the burden on diabetes healthcare teams and capitalize on existing expertise and knowledge. Although funding and sustainability challenges were acknowledged with this approach, drawing on or collaborating with existing site-specific resources was identified as a potential gap that could be addressed. In particular, exploring sustainability and scale-up of BTG will be important to evaluate in future [16]. Offering formal training in QI (e.g., certificate programs in QI) could build skills and knowledge to enhance transition practices and processes further. Integrating formalized training opportunities in QI may incentivize participation and alleviate the burden of content creation.

### Limitations

This study has several limitations. First, it was designed and initiated before and conducted during the COVID-19 pandemic. The disruption caused to healthcare teams across Ontario (not just those involved in pediatric diabetes care) must be acknowledged. The COVID-19 pandemic impacted all healthcare teams and research conduct. As a result, it became difficult to deliver transition care and to track outcomes. While we had initially planned to conduct focus groups with teams at each study site, it became necessary to switch to individual interviews. With short staffing, staff turnover and other disruptions, scheduling individual interviews remotely proved the best way forward. While only 13 interviews were possible, they generated rich and nuanced data, yielding important insights into BTG’s implementation and outcomes. In addition, we only examined practitioners’ perspectives, not those of adolescents living with T1D; future work should aim to engage patients in designing QI AF interventions. It would also have been preferable to have a greater diversity of genders represented among participants (most were women).

## Conclusions

Our study reveals how the BTG study developed a CoP among Ontario practitioners involved in the transition care of young people with T1D. Our findings offer important insights to other investigators planning to implement complex QI studies in other jurisdictions. The CoP could be scaled up to promote a learning health system [32] in pediatric diabetes care provincially and nationally. This may be achieved on a provincial level via the Ontario Pediatric Diabetes Network, a network of 35 pediatric diabetes education programs in Ontario [33]. Ontario is also implementing a provincial pediatric diabetes registry that could be leveraged to facilitate the measurement of transition processes and outcomes and could provide feedback to individual providers and diabetes teams across the province. On a national level, transition-related work could be supported by the CAnadian PediAtric diabetes ConsortIum (CAPACITY), a CIHR-funded initiative to co-develop a national registry to advance pediatric diabetes care and health services research with patients/families, HCPs, researchers, and policymakers. The potential exists for AF data drawn from national and international pediatric diabetes registries to support the development of CoPs. A systematic review of QI interventions demonstrated that such registries have been underused to date [34], but hold great potential for improving care delivery and outcomes internationally.

The BTG CoP suggests other areas where the transition care could be improved, such as establishing provincial or national online transition resources -- for instance, GoT Transition tools, readiness assessment tools, and transition-specific educational resources. As we found in our study, it would be important for transition-related and other QI initiatives to be supported by a CoP to promote engagement, learning, and the sharing of resources.

## Electronic supplementary material

Supplementary Material 1

## Data Availability

Our data cannot be shared openly because of the need to protect the privacy of study participants. The datasets generated and analysed during the current study are not publicly available due to ethical concerns and the importance of safeguarding the identities of our participants. Restricting access to the full interview transcripts is based on concerns regarding identifiability of the participants and the fact that they did not consent to share their data in a publicly available repository. Data are stored securely with the corresponding author. Readers may contact the corresponding author directly with any queries regarding data access.
