# Supplementary material for: Bridging the gap: a qualitative process evaluation from the perspectives of healthcare professionals of an audit-and-feedback-based intervention to improve transition to adult care for young people living with type 1 diabetes
Source: BMC Health Serv Res. 2024 Oct 23;24:1276. doi: 10.1186/s12913-024-11734-1 (PMC11515547; doi:10.1186/s12913-024-11734-1)
Supplement: Supplementary file 1 — Supplementary Material 1 [file 12913_2024_11734_MOESM1_ESM.pdf]

**<u>Interview Guide</u>**

**Guiding questions** [β]

1. Introductions: tell me about your role at your facility.

2. What was it like for you/for your centre to participate in this study?
    a. What aspects of the study did you like?
    b. What aspects of the study as it unfolded were problematic/difficult for your centre?
    c. Could you comment on how the COVID-19 pandemic might have impacted study procedures (and processes) themselves?

3. Now I'd like to hear about specific aspects of your quality improvement (QI) initiative, which involved developing your AIM statements, and carrying out a specific intervention tailored to your own site's specific context and needs.
    a. How did you tailor your QI initiative to your site's transitioning patients?
    b. What are your thoughts about having each site implement their own local QI project as opposed to having each site implement the same initiative (across all sites)?
    c. What impact, if any, did the COVID-19 pandemic have on transition care in your clinical setting?

4. Each centre received feedback reports during the study. We'd like you to share your perspective of the feedback reports you saw/received.
    a. Do you remember receiving this information? Were you able to access the reports?
    b. How did you use the reports?
    c. Were you able to use the reports to inform your QI intervention?
    d. Have you been able to attend any of the webinars? Why/why not?
    e. If you did attend the webinars, what was that like?
    f. Have you accessed Quorum, where the online resources are? Why/why not?

5. Now we want to review the latest feedback report with you. This includes aggregate data across all the study sites compared with data for your site specifically.
    (pause and scroll through to review)
    a. What are your first impressions when you look at these results? Do you have any impressions of how things ran at your site that might explain why they look the way that they do?
    b. Thinking about how the COVID-19 pandemic has impacted transition care in your clinic, how may *that* have impacted these results?

6. Do you have any further thoughts on caring for young people with diabetes and how we can improve their transitions in care?
    a. What are your thoughts/suggestions for 'scaling up' the work of Bridging the Gap?

[β] N.B. These questions served as a guide only, and the participants were free to discuss the topics in any order they wished as the conversation progressed. Each guiding question was followed by further probes, a few of which have been included here.
